# Efficacy of an Education Session by Pharmacists for Patients With Asthma: Protocol and Design of a Randomized Controlled Trial

**DOI:** 10.2196/10210

**Published:** 2018-12-18

**Authors:** Elida Zairina, Gesnita Nugraheni, Gusti NV Achmad, Arie Sulistyarini, Yunita Nita, Arief Bakhtiar, Muhammad Amin

**Affiliations:** 1 Department of Pharmacy Practice Faculty of Pharmacy Universitas Airlangga Surabaya Indonesia; 2 Faculty of Medicine Department of Pulmonology and Respiratory Medicine Universitas Airlangga Surabaya Indonesia; 3 Department of Pulmonology Dr Soetomo Hospital Surabaya Indonesia; 4 Department of Pulmonology Universitas Airlangga Hospital Surabaya Indonesia

**Keywords:** asthma control, education session, pharmacist

## Abstract

**Background:**

Asthma is a chronic disease that requires indefinite long-term therapy. Many approaches have been developed to enable people with asthma to live as normally as possible. In medication therapy management, pharmacists could play important roles in supporting the everyday life of asthmatic patients, such as by providing education therapy management to ensure that patients achieve optimal therapeutic outcomes. A good collaboration between health care practitioners and patients will produce a better system in terms of therapeutic management, which will lead to health care cost savings related to emergency visits. Although the Government has made various efforts to manage asthma in Indonesia, without commitment and support from both patients and health care professionals, the expected outcomes cannot be achieved.

**Objective:**

This study aims to evaluate the effectiveness of an educational intervention provided by pharmacists compared with that of usual care.

**Methods:**

A randomized controlled trial comparing usual care with an education session by pharmacists is underway. The intervention comprises a one-on-one education session of 60 minutes with a pharmacist comprising information regarding (1) asthma medication that has been used; (2) how to use asthma medication devices correctly; (3) asthma symptoms and how to prevent exacerbation of asthma; and (4) how to manage asthma triggers and environmental control measures. The primary outcome measure is change in asthma control, as measured using the Asthma Control Questionnaire. Secondary outcomes include changes in Asthma Quality of Life Questionnaire score, lung function, asthma-related health visits, days off from work or study, and oral corticosteroid use. Research assistants who are masked to the group allocation will collect outcome data at the baseline and every month for a 3-month period. Informed consent will be sought at enrollment and intention-to-treat analysis will be performed.

**Results:**

This study was funded in January 2017 and ethical approval was obtained in June 2017. The enrollment was started in August 2017, and about 72 participants have been enrolled. First results are expected to be submitted for publication in 2019.

**Conclusions:**

This is the first study to evaluate the effectiveness of a pharmacist-guided asthma education session compared with that of usual care in Indonesia. If it is proven effective, this intervention program could improve asthma self-management by patients, which may reduce risks of poorly controlled asthma. This intervention could also be implemented in addition to the current usual care for patients with asthma.

**Trial Registration:**

Thai Clinical Trials Registry TCTR20171219001; http://www.clinicaltrials.in.th/index.php? tp =regtrials&menu=trialsearch&smenu=fulltext&task=search&task2=view1&id=3068 (Archived by WebCite at http://www.webcitation.org/73Ci5eKtv)

**International Registered Report Identifier (IRRID):**

DERR1-10.2196/10210

## Introduction

Asthma is a chronic disease that affects people of all ages worldwide. Uncontrolled asthma can restrict patients’ daily activities and can place them at risk of death. According to the World Health Organization and Global Initiative for Asthma, as many as 300 million people worldwide, of different ages and races, are exposed to asthma; this number is predicted to increase to 400 million by 2025 [1]. Asthma is a health problem in both developed and developing countries [1-3]. Approximately 250,000 people die of asthma each year [3,4]. In 2007, asthma caused 3447 deaths in the United States—equivalent to approximately 9 people each day [5]. Death due to asthma is more common among adults than among children; it is also more common among women (2173) than among men (1274) [5]. In the United Kingdom, >10% of the adults exhibit asthma [6,7]. Although the proportion of population affected by asthma in Asian countries, including Indonesia, is lower than that in Europe or America, the proportion of elderly patients with asthma in Asian countries is quite high (1.3%-15.3%) [8]. Asthma can be controlled with appropriate therapeutic management. Patients with controlled asthma conditions can participate in normal activities and are not likely to experience fatal asthma symptoms [9].

Asthma is a chronic disease that requires indefinite long-term therapy. Various approaches are needed to optimize therapeutic treatments among asthma patients. Several studies in developed countries, such as the United States [10,11] and Australia [12-14], have showed that better health outcomes for asthma patients resulted from the following factors: adequate knowledge among asthma patients, regular monitoring of therapy, and high level of understanding among both health care professionals and patients regarding the disease management behavior. In Indonesia, asthma is one of the top 10 causes of morbidity and death, together with chronic bronchitis and emphysema [15]. In April 2007, observations in 5 provinces of Indonesia (North Sumatra, Central Java, East Java, West Kalimantan, and South Sulawesi) conducted by multiple chronic and degenerative disease subdivisions showed that, in general, asthma control efforts have not been effectively implemented; moreover, there is minimal availability of the equipment required for the diagnosis and management of asthma patients in health care facilities. In 1995, the prevalence of asthma in children was 12%; in 2008, results of the International Study of Asthma and Allergies in Childhood showed that asthma prevalence in 12-14-year-old children was 12.6% [16].

It is currently unknown how the implementation of pharmacist-guided education in asthma self-management might affect asthma self-management in Indonesia as there has been limited research regarding the understanding and behavior of patients for the management of asthma. Therefore, the proposed study has been designed to determine the effectiveness of pharmacist-guided education for asthma patients in Indonesia. Because medications play important roles in successful management of chronic diseases, including asthma, the role of pharmacists’ expertise is essential in the implementation of medication therapy management. Studies have shown that improved asthma control can be achieved if patients are involved in self-management, including self-monitoring of asthma symptoms or lung function, as well as when patients follow written asthma action plans while maintaining regular contact with their health care professionals [17].

This study aims to evaluate the efficacy of pharmacist-guided education sessions provided to patients with asthma compared with that of usual care. We hypothesize that the intervention group will demonstrate superior asthma control, as measured by changes in the Asthma Control Questionnaire (ACQ) scores, after 3 months from the baseline.

## Methods

### Study Setting

Participant recruitment is ongoing at the outpatient Departments of Pulmonology at Universitas Airlangga Hospital. The study has been approved by the human research ethics committee of Universitas Airlangga Hospital. All participants provide written informed consent at the time of enrollment.

### Study Design

**Figure 1:**
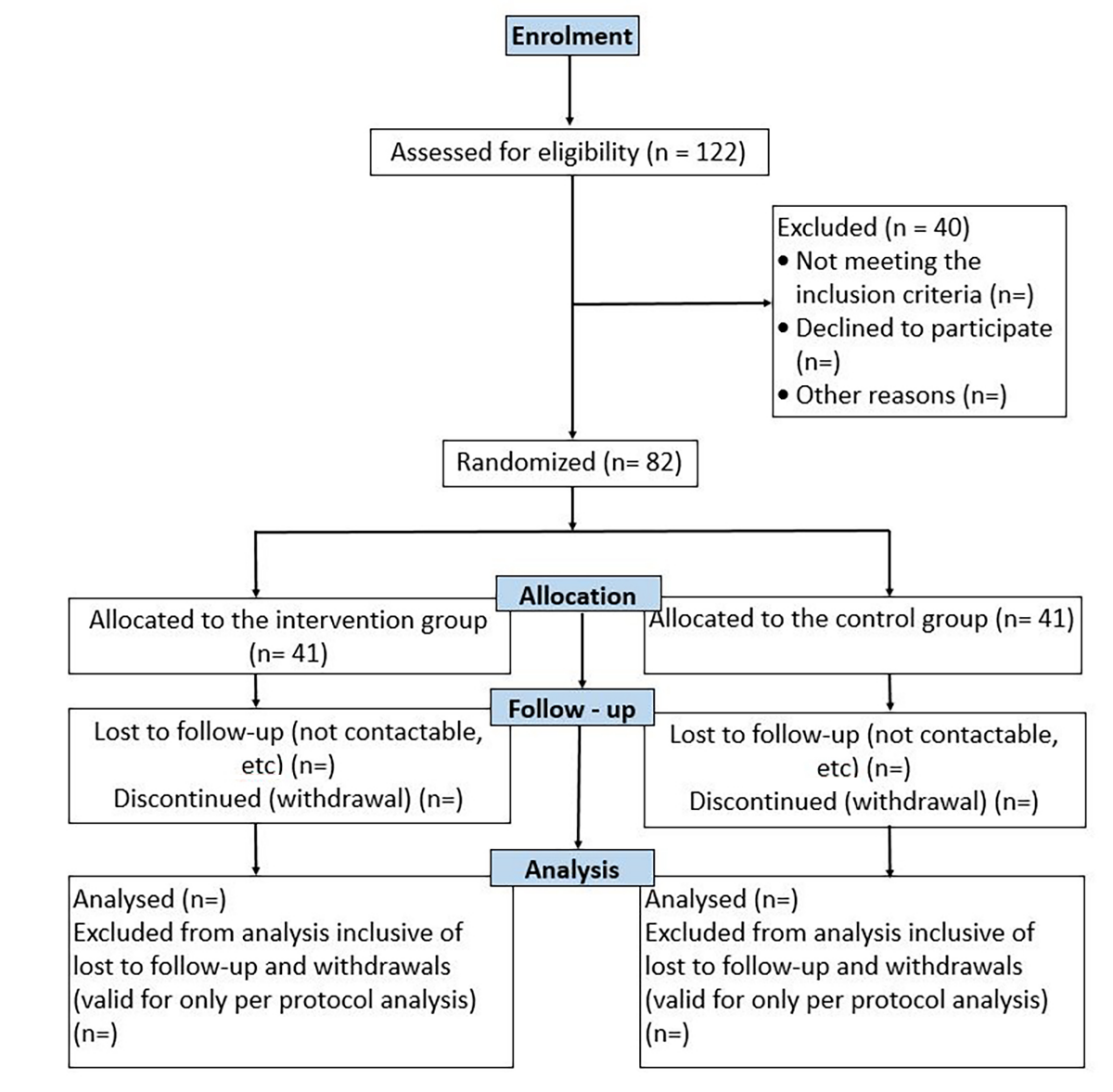
Flowchart of study participants.

This study is designed as a prospective, single-blinded, randomized controlled trial (Thai Clinical Trials Registry # TCTR20171219001); outcome assessors will be masked to group allocation at follow-up assessments. The flow of participants with the expected number is illustrated in Figure 1. The total duration of the study is 3-6 months, depending on the timing of the first visit and patient enrollment in the study. Both groups will be followed up for 3 months; the outcomes will be compared at 1, 2, and 3 months from the baseline to evaluate the efficacy of the intervention.

### Inclusion and Exclusion Criteria

Eligibility for this trial includes patients with asthma who have used any regular medications for asthma within the previous 12 months, who are 18 years of age or older, and are able to communicate in Indonesian. Those who are unable or contraindicated to demonstrate the lung function with spirometer will be excluded.

### Trial Recruitment

The following methods of identification and recruitment of participants will be used in this study: First, the doctors will identify all patients with asthma visiting the outpatient Department of Pulmonology at Universitas Airlangga Hospital on the date of each patient’s clinic visit. The research assistants will approach potential participants and perform screening on the basis of the inclusion and exclusion criteria that were described earlier in this protocol. Then, participant explanatory statements and expression of interest forms will also be provided at the outpatient Department of Pulmonology at Universitas Airlangga Hospital, which has access to the relevant information. Potential participants will be asked to leave their contact details to allow one of the research assistants to contact them. Finally, if a patient agrees to participate, written informed consent will be sought.

### Group Allocation

Recruited participants will be allocated to intervention or control group on a 1:1 basis. Allocation will be concealed using the sealed opaque envelope technique. Random blocks of 4 and 6 will be chosen, and random numbers will be generated using a random allocation software program [18] by an external researcher not involved in the study. Only this researcher will know the allocation sequence. At the time of recruitment, the researchers coordinating this study will open the numbered envelope and allocate each participant to the control (usual care) group or the intervention (education) group. The outcome assessors will be masked to participant group allocation during follow-up assessments.

### Control and Intervention Group

#### Control: Usual Care Group

Participants allocated to the control group will receive usual medical care provided by the Department of Pulmonology and its health care professionals. This includes regular monthly visits, depending on each patient’s asthma severity or complications. If, during follow-up, it becomes apparent that asthma control has deteriorated since the prior assessment (eg, using the asthma reliever ≥3 times per week or requiring an increased preventer dose), the participant and corresponding health professionals will be notified with the participant’s permission.

#### Intervention: Education Group

Before the delivery of educational sessions to patients, pharmacists in this study will undergo training provided by a certified asthma educator (EZ). The trial evaluates an intervention involving pharmacists to deliver a *one-on-one* education session regarding (1) asthma medication that has been used; (2) how to use asthma medication devices correctly; (3) asthma symptoms and how to prevent exacerbation of asthma; and (4) how to manage asthma triggers and environmental control measures. A video that explains how to properly use asthma medications with a variety of devices will be shown to the intervention group. The intervention group will also be provided with an asthma booklet that explains how to correctly use asthma medication and avoid asthma triggers. One of the researchers will contact participants’ health care professionals if any medication changes or unscheduled asthma-related visits are needed.

**Figure 2:**
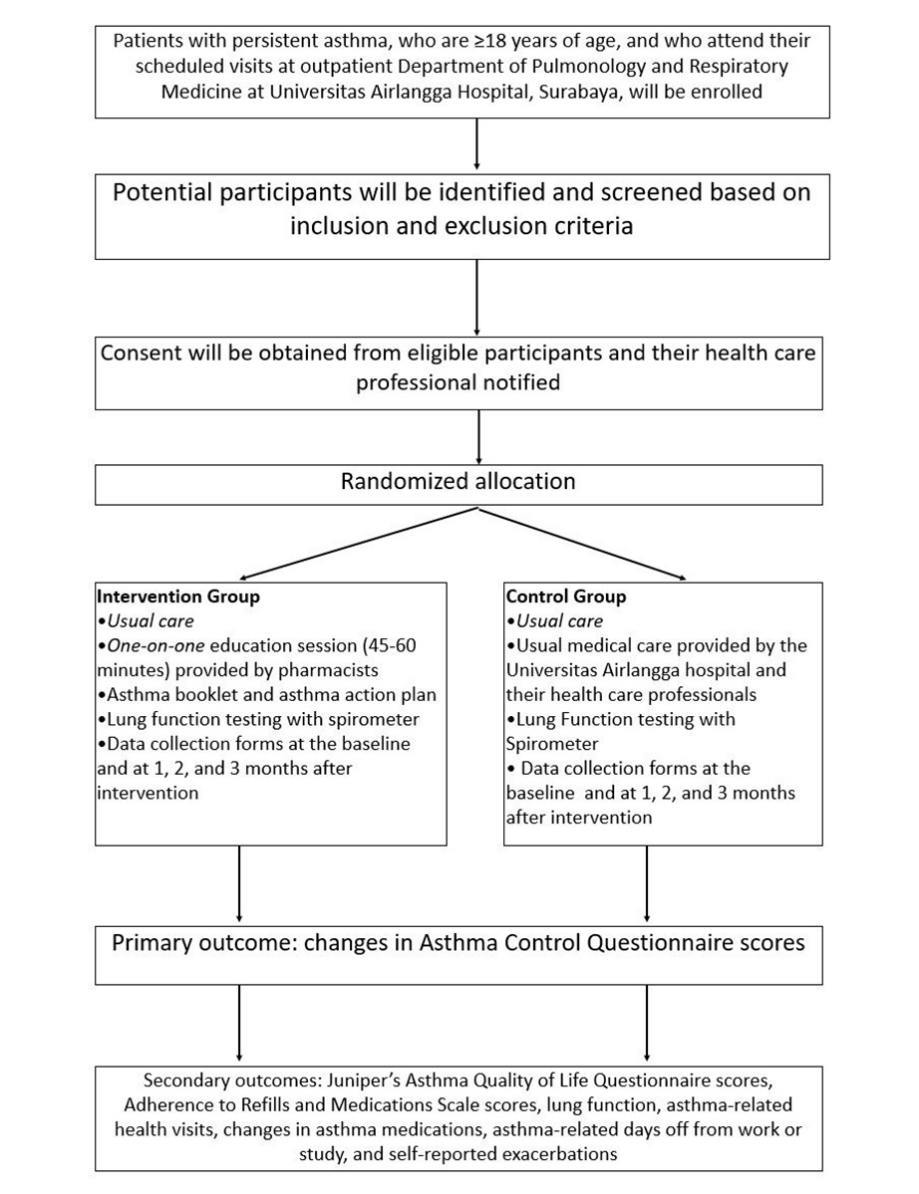
Flowchart of the study.

A written asthma action plan, consistent with the Global Initiative for Asthma guidelines, has been translated into the Indonesian language and will be used to design a participant-specific treatment plan based on the information obtained at the baseline. The asthma action plan contains instructions regarding which medications to take when feeling well, how to recognize worsening asthma, what to do when symptoms are worsening, and what to do in the event of an acute attack, including a first aid plan. The flow of the study is described in Figure 2.

### Outcome Measures

The primary outcome measure is change in asthma control, as measured by the Juniper ACQ [19]. Secondary outcomes include changes in Juniper’s Asthma Quality of Life Questionnaire score [20], lung function, Adherence to Refills and Medications Scales scores, asthma-related health visits, days off from work or study related to asthma, and oral corticosteroid use.

### Data Collection and Follow-Up

ACQ scores, Juniper’s Asthma Quality of Life Questionnaire scores, Adherence to Refills and Medications Scale scores, asthma-related health visits, asthma-related days off from work or study, oral corticosteroid use, and preventer or reliever use data will be collected at the baseline and at 1, 2, and 3 months from the baseline to allow comparisons. Identical data collection forms will be used for both groups. The assessors responsible for collecting outcome data at 1, 2, and 3 months will be masked to participant group allocation.

### Sample Size

A sample size of 28 participants per arm using an estimated SD of 0.66 in ACQ scores will have 80% power (with 2-sided 5% significance level) to detect the minimal clinically important difference in the ACQ score of ≥0.5 between the groups [21,22]. To allow for 25% attrition, 41 participants will be recruited for each arm.

### Data Analysis

The primary analysis will be performed in accordance with the intention-to-treat principle. The baseline characteristics of the 2 groups will be compared using Student’s *t* test for normally distributed continuous variables, Mann-Whitney *U* test for nonnormally distributed continuous variables, and chi-square or Fisher’s exact test (as appropriate) for categorical variables. Primary inferential analysis will be conducted using a mixed effects model for the intention-to-treat population. This model will include the treatment group and time as fixed effects, with an interaction between treatment and time to ascertain if the groups behave differently over time. Other demographic and clinical factors will be included as potential covariates in the mixed effects model. Comparisons will also be made of the following: the proportion of participants whose ACQ score improves >0.5 (minimal clinically important difference) over the study period, the proportion in whom asthma remains “not well controlled” (ACQ score ≥1.5), and those whose asthma is “well controlled” (ACQ score <1.5) at each time point [23]. Secondary outcomes will be summarized using descriptive statistics; analyses will be performed using the methods described above.

## Results

This study was funded in January 2017 and ethical approval was obtained in June 2017. The enrollment was started in August 2017 and is currently ongoing; we plan to complete the enrollment by December 2018. About 72 participants have been enrolled in the trial. First results are expected to be submitted for publication in 2019.

## Discussion

This is the first study to evaluate the effectiveness of a pharmacist-guided asthma educational session compared with that of usual care in Indonesia. If it is proven effective, this intervention program could improve asthma self-management by patients, which may thus reduce the risk of poorly controlled asthma. This intervention could also be implemented along with the current usual care for patients with asthma. This trial is designed to evaluate an educational program for patients with asthma in order to enable better self-management for the control of their asthma. The individualized written asthma action plan designed for each patient provides clear guidelines in terms of actions to be taken in case of worsening asthma.

The proposed intervention has the potential to improve asthma outcomes by facilitating better asthma self-management. This may translate to reduced health care costs in the form of fewer asthma-related unplanned medical and emergency department visits. If the intervention is efficacious, this could potentially influence clinical practice and health policy.

In order to improve health outcomes of asthmatic patients, solutions include regular monitoring as well as education regarding medication use and compliance. Most chronic diseases require patients to undergo therapy for an indefinite duration to meet therapeutic goals. Pharmacists have an important role in ensuring patients achieve optimal therapeutic outcomes. A pharmacist should be aware of this role, which involves providing adequate information to patients with chronic diseases. By building collaborations among health care professionals, a robust system of therapeutic management for patients with asthma will be established, enabling improvement of health outcomes and potentially reducing health care costs. Multiple efforts have been initiated by the government to manage asthma in Indonesia; however, without support and commitment from both health care professionals and community, the desired results cannot be achieved.

## Abbreviations

ACQ: Asthma Control Questionnaire
